# System of Medicine

**Published:** 1885-02

**Authors:** 


### "SYSTEM OF MEDICINE."

The Hahnemann Publishing House has in press the first volume of their *System of Medicine*. Of the scope of this work they speak as follows:

"The general object in view is to present to the homœopathic practitioner a work on Practice which will embody not only sound homœopathic therapeutics, but shall give reliable and extensive chapters on ætiology, pathology, etc., and shall discuss, in a pointed and practical way, the various means of physical diagnosis, *auxiliary* measures in the treatment of the sick, preventive medicine—in short, shall, with homœopathic treatment as presented in former works of our school, give to the homœopathic student and practitioner that special information which has been rather meagrely furnished heretofore in our literature."

